# Supplementary material for: Complexome profiling of the Chlamydomonas psb28 mutant reveals TEF5 as an early PSII assembly factor
Source: Plant Cell. 2025 Mar 17;37(6):koaf055. doi: 10.1093/plcell/koaf055 (PMC12164590; doi:10.1093/plcell/koaf055)
Supplement: koaf055_Supplementary_Data [file koaf055_supplementary_data.zip › psb28_interactive.zip!/plots/intensity/qualityPlots/peptides/CIS2_Cre03.g149100.t1.2.html]

### Cre03.g149100.t1.2

Trivial name: CIS2  
SeqCoverage [median]: 43.00  
Mass: 54.53 kDa  
  
 

| Sequence | MissedCleavage | Mass [Da] | wtIntensity | muIntensity | Score |
| --- | --- | --- | --- | --- | --- |
| AGGDGVGLR | 0 | 800.41 | 24454.12 | 46115.06 | 183.26 |
| ALQDAALSDEYFVSR | 0 | 1683.81 | 14828.34 | 23129.26 | 204.13 |
| ALQDAALSDEYFVSRK | 1 | 1811.91 | 404.61 | 0.00 | 29.73 |
| EMQDKQIVR | 1 | 1145.59 | 0.00 | 438.40 | 43.08 |
| ESLTDPDTK | 0 | 1004.47 | 12947.03 | 12195.96 | 125.16 |
| GTLTVVDNR | 0 | 973.52 | 19242.87 | 29202.94 | 144.12 |
| HYSDVVVR | 0 | 973.50 | 1237.33 | 805.22 | 88.94 |
| IGSVENIPAFIAGVK | 0 | 1513.85 | 9142.49 | 0.00 | 193.15 |
| IIKDVAENEVFPLVGVDPLIEIAK | 1 | 2620.47 | 3087.43 | 0.00 | 76.20 |
| IIRPQQDYK | 1 | 1159.63 | 20979.08 | 30107.80 | 96.14 |
| IPTIAAMAYHK | 0 | 1214.65 | 9492.80 | 5631.08 | 81.02 |
| ISFIDGDKGILR | 1 | 1332.74 | 21852.20 | 64323.86 | 72.96 |
| KLYPNVDFYSGLVYR | 1 | 1832.95 | 4000.62 | 0.00 | 88.73 |
| KYTLEISDGGTINAQALK | 1 | 1921.02 | 0.00 | 2574.36 | 70.33 |
| LLGKIPTIAAMAYHK | 1 | 1625.93 | 0.00 | 1513.01 | 68.83 |
| LPPSNAYNR | 0 | 1030.52 | 0.00 | 648.85 | 75.85 |
| TAEGADTLSK | 0 | 991.48 | 39054.39 | 46221.00 | 163.16 |
| TYDPGYTNTTAVISR | 0 | 1657.79 | 13301.88 | 19316.67 | 169.89 |
| YRGYPIEELAAR | 1 | 1436.74 | 2963.18 | 8545.24 | 76.52 |
| YTLEISDGGTINAQALK | 0 | 1792.92 | 447.16 | 0.00 | 113.61 |
|  |  |  | 197435.5 | 290768.7 |  |
